# Design rules for adhesion-driven synthetic cell motility on dynamic membranes

**DOI:** 10.1039/d5sc05324b

**Published:** 2025-08-19

**Authors:** Daniele Di Iorio, Ali Heidari, Seraphine V. Wegner

**Affiliations:** a Institute of Physiological Chemistry and Pathobiochemistry, University of Münster Münster Germany diiorio@uni-muenster.de wegnerse@uni-muenster.de

## Abstract

Cell motility is a fundamental process involved in many complex cellular events and the development of synthetic cells that mimic cell motility enables us to understand the composite mechanisms underlying it. Here, we use giant unilamellar vesicles (GUVs) and supported lipid bilayers (SLBs) as simplified models to investigate how the surface density of ligands and their lateral mobility influences adhesion-dependent cell motility. In particular, we use the photoswitchable interactions between the proteins iLID (improved light-inducible dimer) and nano (wild-type SspB) to induce light-responsive adhesions of the GUVs on the SLBs and systematically tune adhesion properties by varying receptor and ligand densities, and assess their effects on the reversibility and dynamics of adhesion. We find that ligand mobility, while essential for dynamic interactions, can lead to ligand-receptor clustering that disrupts adhesion asymmetry and limits directional motility. Conversely, high ligand densities restrict mobility, enabling adhesion asymmetry and GUV migration upon localized illumination but at the cost of reduced reversibility. These results define a design space in which both ligand mobility and density must be finely balanced to achieve reversible, light-guided motility. Our findings provide fundamental insight into adhesion-based migration mechanisms on dynamic membranes and establish design principles for engineering synthetic cells capable of controlled, directional movement on dynamic substrates.

## Abstract 2

We report how both the lateral mobility and surface density of ligands on lipid surfaces influence the ability of synthetic cells to maintain a light-induced directional motility.

## Graphical abstract

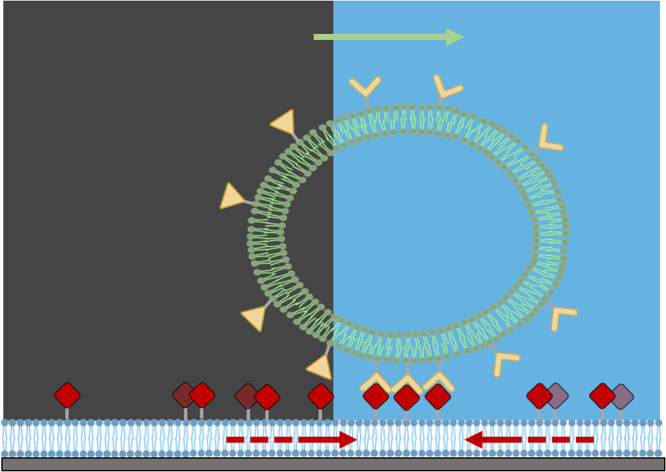

## Introduction

Cells exhibit the remarkable ability to migrate through diverse environments, adapting to a wide range of chemical and physical conditions.^1,2^ Over the course of evolution, cells have developed multiple mechanisms to achieve movement, and can even switch between different migration modes depending on the context. Cellular motility spans a spectrum of strategies, including adhesion-based mesenchymal migration along extracellular matrices, ameboid migration driven by internal cytoskeletal dynamics, and solution-based motility propelled by flagellar movement.^3^ Recreating these modes of motility in synthetic systems, also called synthetic cells, offers a powerful approach to understand mechanisms underlying cell migration.^4^ These reductionist systems allow us to probe the physical and biochemical principles that govern the limits and capabilities of various motility strategies, while also enabling the design of mobile, cell-sized synthetic objects for diverse applications.

Inspired by the different type of cellular motility mechanisms, several approaches have been reported to implement motion in minimal synthetic cells.^5^ Recent studies have demonstrated that nanoparticles and lipid vesicles can be equipped with protein and DNA-based machinery to mimic different aspects of cell motility.^4^ For instance, encapsulating actin filaments^6,7^ or microtubules^5,8^ inside giant unilamellar vesicles (GUVs) has been shown to induce shape changes and ameoboid-like movement upon filament polymerization. To mimic cell motility in free solution, synthetic vesicles and nanoparticles have been propelled by external fields,^9^ Marangoni flows,^10,11^ and enzymatic reactions.^12,13^ Alternatively, DNA-based walkers offer another strategy, moving 20–200 nm particles along predetermined paths through adhesion-dependent strand displacement reactions.^14^ Collectively, these approaches illustrate the richness of mechanisms available for engineering synthetic motility across various environments and length scales.

In adhesion-based cell migration in eukaryotic cells, movement is driven by coordinated events: protrusions at the leading edge forming lamellipodia, formation of new adhesions to the extracellular matrix in the front, myosin-dependent contraction of the cell body, and detachment of adhesions at the trailing-edge.^1,3,15^ This dynamic and synchronized sequence of events is tightly regulated over multiple time and length scales by adhesion receptors, cytoskeletal linkers, and signaling molecules that asymmetrically distribute within the cell. Moreover, both the spatial organization and mobility of adhesion ligands in the extracellular environment influence migration and the cell can trigger ligand-receptor clustering as well as ligand reorganization.^16,17^ In fact, ligand clustering is known to enhance cell migration in some cases,^16^ and ligand mobility has been shown to significantly affect cell spreading, cytoskeletal organization, and signal activation.^18^

Efforts to mimic adhesion-based migration have reproduced these features in synthetic cells.^19,20^ Notably, dynamic protein-mediated interactions have been established between giant unilamellar vesicles (GUVs, 10–50 μm) and substrates, introducing the asymmetry in adhesions similar to a migrating cell.^21^ One strategy employed the traveling wave patterns generated by MinD and MinE proteins on a 2D substrate to drive the continuous motion of GUVs through a direct mechanochemical feedback loop.^22^ In previous work from our group, we demonstrated light-guided, directional movement of GUVs using photoswitchable adhesions.^21^ By employing the interaction between iLID (improved light-inducible dimer, based on the LOV2 domain) and its binding partner micro (SspB R73Q), where iLID binds to micro under blue light and dissociates from it in the dark, we created reversible, asymmetric adhesions between the front and rear of migrating GUVs. Despite these advances, the role of receptor and ligand mobility and their clustering remains poorly understood in the context of adhesion-based synthetic cell motility. This is particularly relevant when considering receptor mobility within the plasma membrane or cell motility on dynamic substrates and on top of other cells, where the adhesion ligands can rearrange.

In this work, we aim to understand how the lateral mobility and surface density of ligands affect the ability of GUVs to maintain directional movement, how ligand-receptor clustering contributes to adhesion dynamics, and particularly how this mobility can counteract the reversibility of adhesions and disrupt the required asymmetry in adhesions. To address these questions, we employed supported lipid bilayers (SLBs) as model substrates that allows adhesion ligands to diffuse in two dimensions, while GUVs move on top of the SLBs. By systematically tuning the densities of ligands and receptors on the SLB and GUV membranes, we identify the parameters necessary for effective GUV motility on mobile substrates and define the limits at which adhesion-based migration fails.

## Results and discussion

### Correlation between receptor density and receptor mobility on SLBs

Building on our previous work, where we demonstrated light-guided motility of GUVs through the formation of asymmetric, photoswitchable adhesions,^21^ we now investigate how ligand mobility influences this process. In our earlier setup, iLID was immobilized on a PEG-coated glass substrate and micro was anchored to the GUV membrane. Partial illumination of the GUVs triggered adhesions at the front, driving motion toward the illuminated area. Importantly, these adhesions were reversible in the dark, enabling directional movement by spatially modulating the illumination area. Moreover, the ligands on the PEG-coated substrate were immobile and could not cluster.

**Fig. 1:**
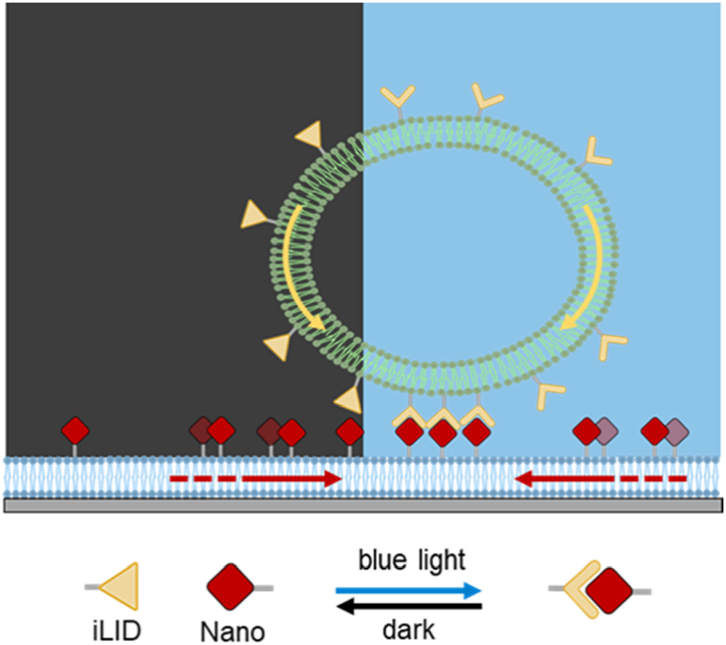
Schematic representation of the light-triggered motility of a giant unilamellar vesicle (GUV) on a supported lipid bilayer (SLB). GUVs decorated with iLID interact with nano-functionalized SLBs. The light-dependent interaction between iLID and nano enables the adhesion of GUVs to the SLBs under blue illumination, thus leading to the movement of the GUV towards the illuminated area.

To address how ligand mobility impacts on the GUV motility, here we employed SLBs as fluid substrates capable of presenting laterally mobile adhesion ligands. SLBs, like GUVs, mimic key features of cellular membranes, including two-dimensional lateral fluidity and the dynamic rearrangement of membrane components.^23,24^ Their lipid composition can be finely tuned to modulate the mobility and density of anchored molecules, making them ideal for probing the role of ligand mobility and density in synthetic cell motility.^25^ In particular, here we immobilized the adhesion ligand nano (wild-type SspB) on SLBs and functionalized the outer surface of GUVs with iLID as the photoswitchable adhesion receptor, forming a reversible receptor–ligand pair that can be triggered with blue light (Fig. 1). Nano is similar to micro but with a different affinity to iLID, and it is generally more employed in the photoregulation of a variety of cellular activity.^26–28^ Both SLBs (DOPC with 0.5–10% DGS-NTA) and GUVs (POPC, 10% POPG and 0.1–0.5% DGS-NTA) contained lipids with Ni^2+^-loaded nitrilotriacetic acid head groups (DGS-NTA) to enable binding of His-tagged proteins.^29^ The DGS-NTA content served as a controllable parameter to vary the surface density of immobilized protein.

**Fig. 2:**
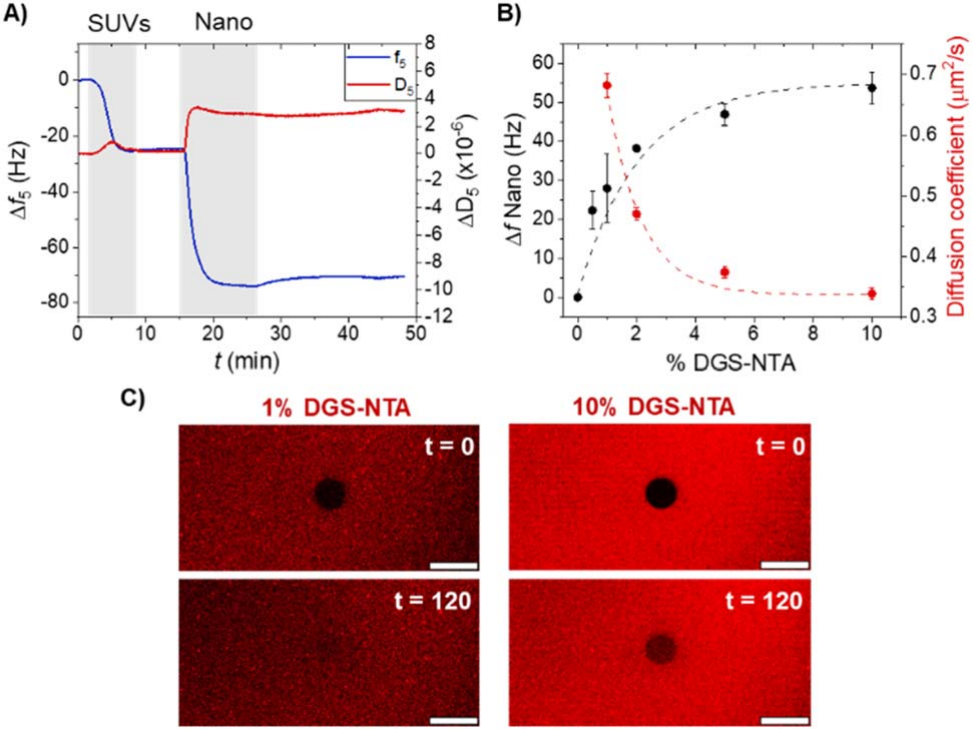
(A) QCM-D measurement showing stepwise the formation of an SLB using DOPC-based SUVs doped with 5% of DGS-NTA lipids, and the subsequent binding of His-tagged nano (1 μM). Gray shadings indicate the addition of the indicated components, while white areas indicate the buffer addition. (B) Limiting frequency shifts obtained for the binding of nano in QCM-D measurements (in black) and the diffusion coefficient of mOrange-nano (in red) as a function of the DGS-NTA fraction in the SLBs. Black and lines are guides to the eye. (C) Fluorescence recovery after photobleaching (FRAP) images of DOPC-SLBs with 1% (left) and 10% (right) DGS-NTA functionalized with mOrange-nano before bleaching (top) and after 120 s recovery (bottom). Scale bar represents 20 μm.

To characterize nano binding to SLBs with different DGS-NTA content (0.5–10%), we first monitored SLB formation and subsequent protein attachment using quartz crystal microbalance with dissipation monitoring (QCM-D). This lipid composition resulted in DGS-NTA lipid densities ranging between 1.15 and 23.1 pmol cm^−2^ (assuming a lipid footprint of 72 A^2^).^29^ QCM-D enables the monitoring of SLB formation and the subsequent protein immobilization steps in real-time, where surface binding events are visible as the decrease of resonance frequency. SLBs were generated by flushing small unilamellar vesicles (SUVs) over SiO_2_-coated QCM-D crystals, resulting in a typical frequency shift (Δ*f*) of 24 ± 1 Hz (Fig. 2a and S1).^30^ Upon flushing an excess of His_6_-tagged nano protein (1 μM) over the QCM-D sensor, a further frequency decrease was observed due to protein binding to the SLB. This decrease in frequency remained stable upon washing with the buffer, indicating a stable protein immobilization (Fig. 2a). Here, the multivalent NTA-His_6_ interactions ensure slow protein desorption kinetics (*t*_1/2_ up to 12 h),^29^ resulting in a steady lipids functionalization throughout the whole experimental timescale. In the nano immobilization step, the change in frequency, which is proportional to the mass bound to the SLB, corresponded to the DGS-NTA content (Fig. 2b and S1). A linear correlation between DGS-NTA content and nano coverage was observed up to 2%, beyond which the surface approached saturation, as minimal additional binding was observed at 5% and above.

The protein surface coverage of SLBs significantly influences the lateral mobility of the proteins themselves. To assess the mobility of nano on SLBs at varying surface densities, we performed fluorescence recovery after photobleaching (FRAP) assays using confocal microscopy. For these experiments, nano was fused to the fluorescent protein mOrange (His_6_-mOrange-nano) for visualization, and this fusion protein was used to functionalize the SLBs. In agreement with the QCM-D results, the fluorescence intensity of immobilized mOrange-nano increased with the DGS-NTA content in the SLB (Fig. S2A and B). As a control, we first confirmed the fluidity of the SLBs by bleaching the DiD dye incorporated in the lipid mixture (Fig. S3). The rapid recovery of DiD fluorescence in the bleached area (*t*_1/2_ = 13.5 s; diffusion coefficient of 0.82 μm^2^ s^−1^), confirmed the successful formation of fluid SLBs in each case. We then measured the diffusion dynamics of mOrange-nano on SLBs containing 1–10% DGS-NTA. FRAP measurements at lower DGS-NTA concentrations (<1%) were not feasible due to insufficient fluorescence signal from the bound protein. The results showed that both the mobile fraction and diffusion coefficient of mOrange-nano decreased with increasing DGS-NTA content (Fig. 2b and S2B). At low protein density (1% DGS-NTA), mOrange-nano exhibited relatively high mobility, with a diffusion coefficient of 0.68 μm^2^ s^−1^. However, at DGS-NTA content above 5%, the diffusion coefficient dropped significantly to 0.33 μm^2^ s^−1^, coinciding with the surface saturation observed in QCM-D experiments (Fig. 2b). This reduction in protein mobility at higher densities is consistent with increased steric hindrance and crowding among the surface-bound proteins, which limits their ability to diffuse laterally. Together, these measurements define two regimes of SLB functionalization: one characterized by low ligand density and high mobility (1% DGS-NTA), and another by high ligand density and reduced mobility (10% DGS-NTA).

### Light-induced adhesions of GUVs on nano-functionalized SLBs

After establishing the relationship between ligand density and mobility on SLBs, we next investigated the light-induced adhesion of GUVs on these surfaces. In these experiments, we chose SLBs containing either 1% or 10% DGS-NTA. The GUVs were functionalized with His_6_-tagged iLID as the photoswitchable adhesion receptor and a membrane dye (DiD) was added for visualization. To tune receptor density, we incorporated 0.1%, 0.25%, or 0.5% DGS-NTA lipids into the GUV membranes, yielding Ni^2+^-NTA head-group surface densities of approximately 0.25, 0.62, and 1.25 pmol cm^−2^, respectively, based on a lipid footprint of 60 A^2^.^31^ Following functionalization with iLID, the GUVs were osmotically deflated by partial evaporation of the outer buffer. This deflation step was essential to generate excess membrane area, enhancing membrane fluctuations^32^ and facilitating new iLID-nano interactions at the GUV-SLB interface.

**Fig. 3:**
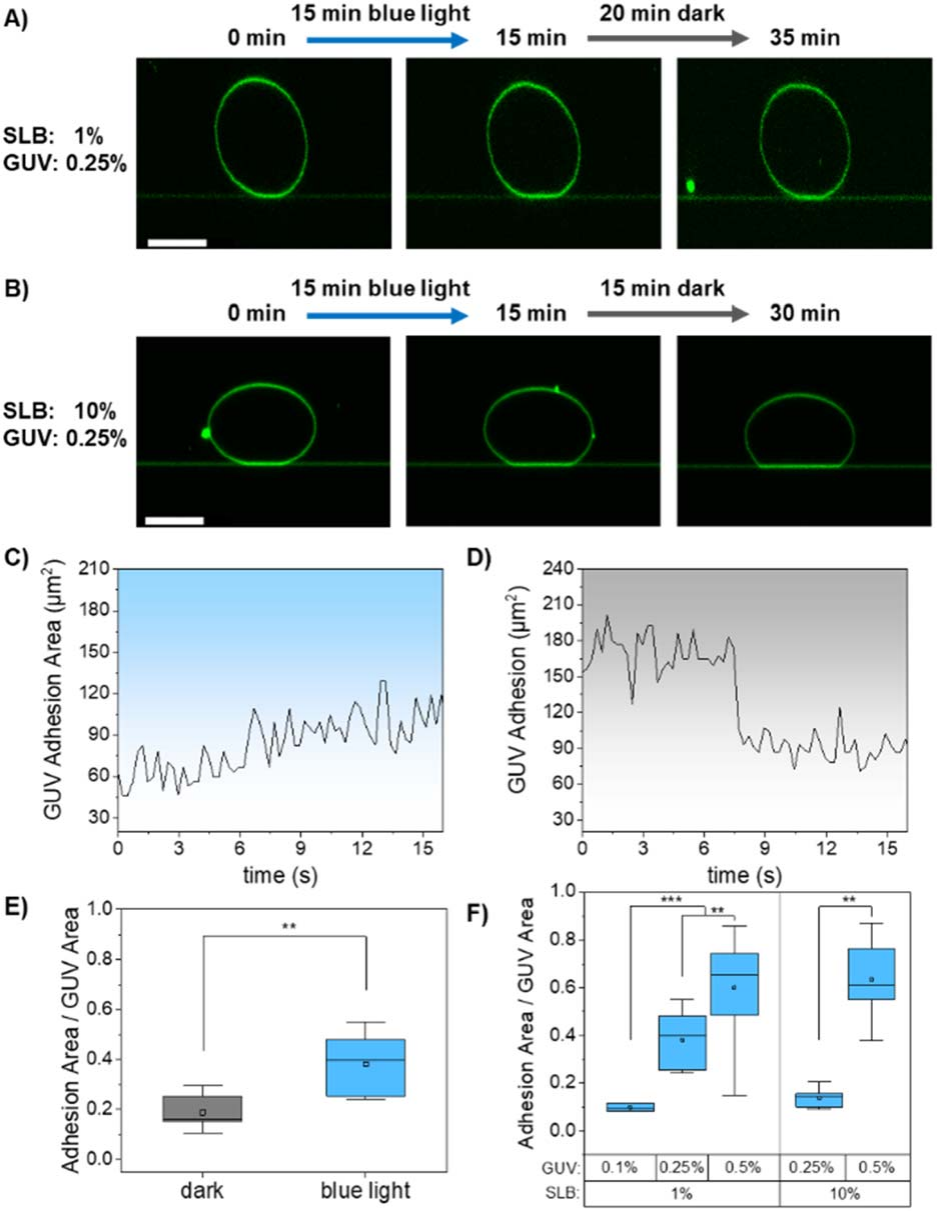
Light-dependent adhesion of GUVs to SLBs. Fluorescence microscopy images of GUV containing 0.25% DGS-NTA and functionalized with iLID adhering on SLBs doped with 1 mol% (A) or 10 mol% (B) DGS-NTA and functionalized with nano. (C) and (D) Time-dependent adhesion area of 0.25% GUV on 1% SLB under blue light illumination (C) and in the dark (D). (E) Normalized adhesions for iLID-GUVs containing 0.25% DGS-NTA on SLBs doped with 1% DGS-NTA in the dark and after blue light illumination. Values represent ratios between the adhesion area of GUVs at the SLBs and the area of the same GUVs at the central section. (F) Normalized adhesion area values for GUVs functionalized with varying iLID densities (0.1, 0.25, and 0.5% DGS-NTA lipid) at different nano densities on the SLBs (1 and 10% DGS-NTA lipid). Each box plot represents the average of 6–10 different GUVs per measurement. *P*-values <0.001 are represented as *** and *p* < 0.01 as **.

We first tested the adhesion behaviour of GUVs with 0.25% DGS-NTA (intermediate receptor density) on nano-functionalized SLBs containing 1% DGS-NTA (low ligand density, high mobility). In the dark, no significant adhesion was observed. Upon exposure to blue light (laser 488 nm for 15 min), the adhesion area increased markedly, reflecting the activation of iLID and its binding to nano on the SLB (Fig. 3A and S4). This adhesion was reversible; following 20 min in the dark, the adhesion area decreased, returning approximately to the pre-illumination state. The time-dependent analysis of this process showed that the adhesion area increased linearly, reaching approximately 1.5 times the initial area within 15 min of blue light illumination (Fig. 3C). When the light was turned off, the adhesion area decreased in a stepwise fashion at around 7 min to baseline (Fig. 3D). Control experiments confirmed that no adhesion occurred in the absence of nano on the SLBs (Fig. S5), validating the specificity of the iLID–nano interaction. To quantify the light-induced enhancement of adhesion, we compared iLID-GUVs exposed to blue light with those kept in the dark for 30 minutes. The adhesion area was normalized to the GUV's cross section (center) to account for variations in vesicle size. Under blue light, GUVs exhibited a twofold increase in adhesion area compared those kept in the dark (Fig. 3E). Furthermore, no relevant correlation was observed between the GUV size and the adhesion to the surface.

To investigate the effect of receptor density on the GUV adhesion, we placed iLID-GUVs with 0.1% (low receptor density) or 0.5% (high receptor density) DGS-NTA on nano-functionalized SLBs with 1% DGS-NTA. GUVs with low receptor density (0.1 mol% DGS-NTA) failed to adhere to these SLBs, even after 15 minutes of blue light illumination (Fig. S6A). In contrast, GUVs with high receptor density (0.5 mol% DGS-NTA) exhibited light-dependent adhesions, which were no longer reversible after returning to dark conditions for 20 minutes (Fig. S6B). This stronger adhesion was reflected in a larger adhesion area for GUVs containing 0.5 mol% DGS-NTA, compared to those with 0.25 mol% (Fig. 3F), highlighting the correlation between receptor density and adhesion strength. To understand the lack of reversibility, it is important to note that although the iLID-nano interaction is quickly and significantly weakened in the dark (*t*_1/2_ = 18 s, *K*_d,lit_ = 0.130 μM, *K*_d,dark_ = 4.7 μM), residual binding can still occur.^33^ Moreover, when iLID was immobilized on an SLB, the affinity to nano changed only approximately four-fold between blue light and dark conditions.^34^ As a result, under circumstances in which light-induced clustering of nano and iLID at the adhesion site takes place, the number of multivalent binding events significantly increases, which generate sufficient adhesion energy to stabilize the attachment and kinetically impedes GUV detachment—even after the light is turned off and the affinity between iLID and nano decreases.

In a second step, we evaluated GUV adhesion on SLBs containing 10% DGS-NTA, representing the high ligand density and low mobility regime. In this case, GUVs containing 0.25% DGS-NTA exhibited light-dependent adhesion (Fig. 3B); however, unlike on the 1% DGS-NTA SLBs, this adhesion was irreversible once illumination was stopped. In fact, some GUVs continued to show increasing adhesion even after the light was turned off, suggesting that receptor mobility also plays a role in regulating the reversibility in this case. Similarly, GUVs with 0.5% DGS-NTA adhered strongly to the high-density SLBs and exhibited even larger adhesion areas than those with 0.25% DGS-NTA (Fig. 3F and S7). As expected, also in this case the adhesion was light-dependent but irreversible in the dark, consistent with the formation of dense multivalent interactions at the GUV-SLB interface. Overall, we observe that both the receptor and the ligand density on the lipid membranes are key parameters that determine if light induced adhesions occur and if they are reversible (Table 1).

**Table 1:** Correlation between light-induced adhesion and adhesion reversibility in the dark of GUVs onto SLBs at varying DGS-NTA densities. Reversion of adhesions is assessed after 20 min in the dark

GUV	0.1%	0.25%	0.5%
SLBs 1%	No adhesion	Reversible adhesion	Irreversible adhesion
SLBs 10%	No adhesion	Irreversible adhesion	Irreversible adhesion

**Fig. 4:**
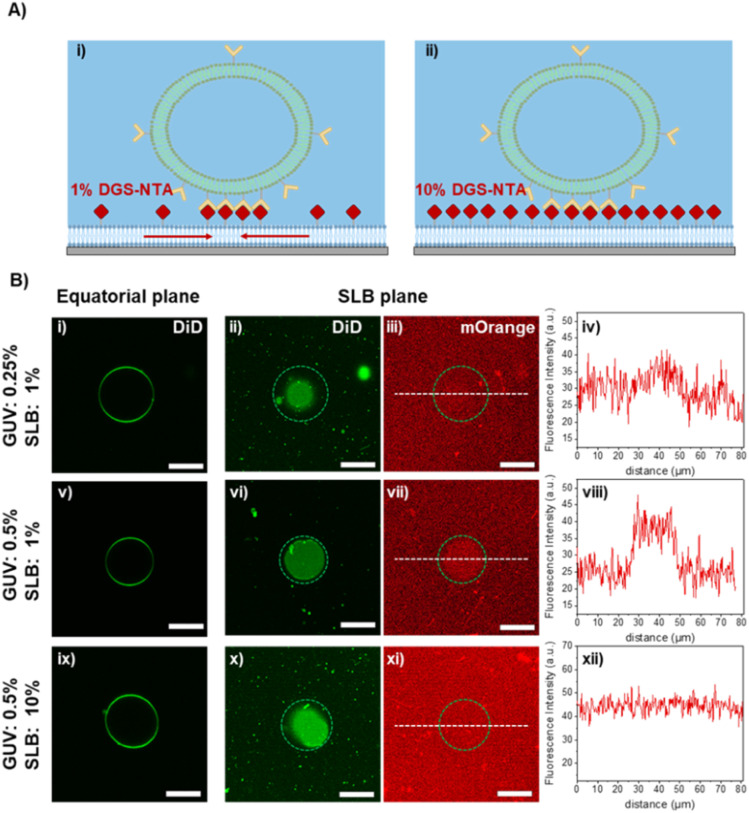
(A) Schematic representation of the light-dependent adhesion of GUVs on SLBs at different ligand densities. At lower ligand densities on SLBs the mOrange-nano proteins clusters at the contact area with the GUVs, while the high density and low mobility of ligands hinders the clustering effect. (B) Fluorescence microscopy images of iLID-functionalized GUVs (with 0.25 and 0.5% DGS-NTA) on nano-mOrange functionalized SLBs (with 1 and 10% DGS-NTA) obtained at different focal planes. The left column shows the equatorial plane of the GUVs; the middle columns show the bottom of GUV at the adhesion area with SLB, and the mOrange-nano layer and the protein recruitment at the adhesion area. The right columns show the fluorescence intensity profiles of mOrange measured in the ROI (white lines). Scale bars indicate 20 μm.

Part of the observed irreversibility in GUV adhesions may result from ligand-receptor clustering and enrichment in the adhesion area due to their lateral mobility upon light-induced binding (Fig. 4A). To assess whether ligands are enriched within the GUV-SLB adhesion zone, we employed fluorescently labelled mOrange-nano ligands at different densities on the SLBs. iLID-GUVs were then placed on these SLBs and incubated under continuous blue light illumination for 30 minutes to ensure maximal adhesion to the mOrange-nano SLBs. To assess ligand clustering, we acquired confocal fluorescence microscopy images of the GUVs at both the equatorial plane and the contact area with the SLBs. Notably, iLID-GUVs containing 0.25% and 0.5% DGS-NTA, placed on SLBs with mobile ligands (1% DGS-NTA), exhibited a clear enrichment of mOrange fluorescence within the contact area (Fig. 4B). In particular, the mOrange signal intensity in the contact area was 1.1-fold and 1.4-fold higher than outside the contact area for 0.25% and 0.5% DGS-NTA, respectively, confirming the occurrence of light-induced ligand-receptor clustering. This enrichment is attributed to the lateral mobility of mOrange-nano within the SLB, allowing it to diffuse and accumulate at the adhesion site, thereby reinforcing the interaction with iLID on the GUVs. In contrast, GUVs with 0.5% DGS-NTA placed on SLBs with low ligand mobility (10% DGS-NTA) did not show any enrichment of the mOrange signal in the adhesion area (Fig. 4D). Interestingly, when the position of iLID and nano was switched (*i.e.*, GUVs with 0.5% DGS-NTA were functionalized with mOrange-nano and the SLBs with 10% DGS-NTA were functionalized with iLID), we observed an enrichment of the mOrange-nano signal on the GUV in the adhesion area (Fig. S8). This further shows that both the ligand on the SLBs and receptors on the GUVs contribute to receptor-ligand clustering. The ligand-receptor clustering results are in agreement with the observed light-dependent adhesion behaviour of GUVs on SLBs with varying ligand densities and help explain the emergence of irreversible adhesion on surfaces with low ligand density but high ligand mobility. Specifically, for SLBs containing 1% DGS-NTA, GUV adhesion leads to the local accumulation of ligands in the contact area, effectively mimicking the ligand density found on SLBs with significantly higher DGS-NTA content. The stronger clustering effect observed with GUVs containing 0.5% DGS-NTA compared to those with 0.25% DGS-NTA suggests that receptor density on the GUV membrane also plays a crucial role in driving ligand enrichment. Notably, receptor clustering is accompanied by a larger contact area in the deflated GUVs as they bind to the SLBs. Taken together, these findings highlight that effective ligand recruitment, requires not only ligand mobility but also a critical threshold of receptor density on the GUV membrane.

### Light-induced motility of GUVs on SLBs

Finally, we explored the motility of iLID-GUVs on nano-functionalized SLBs under varying receptor and ligand densities. Our goal was to induce asymmetric adhesion in a single GUV through partial illumination, such that the GUV would preferentially adhere to the illuminated region and move in that direction. Furthermore, by moving the illuminated region, we aimed to trigger successive steps in GUV movement through the reversal of the formed adhesions at the rear. For these experiments, it was critical to select conditions where light dependent differences are observed and adhesions can be reversed in the dark for guiding the GUV over the surface with light. Based on prior results, we selected GUVs functionalized with 0.25% DGS-NTA placed on SLBs containing 1% DGS-NTA, as these conditions exhibited the reversible light-dependent adhesion behaviour. Upon partial illumination of these GUVs (approximately 30–50% of their adhesion area) using a defined region of interest (ROI, light-red square), we did not observe significant movement, even after 20 minutes (Fig. S9).

**Fig. 5:**
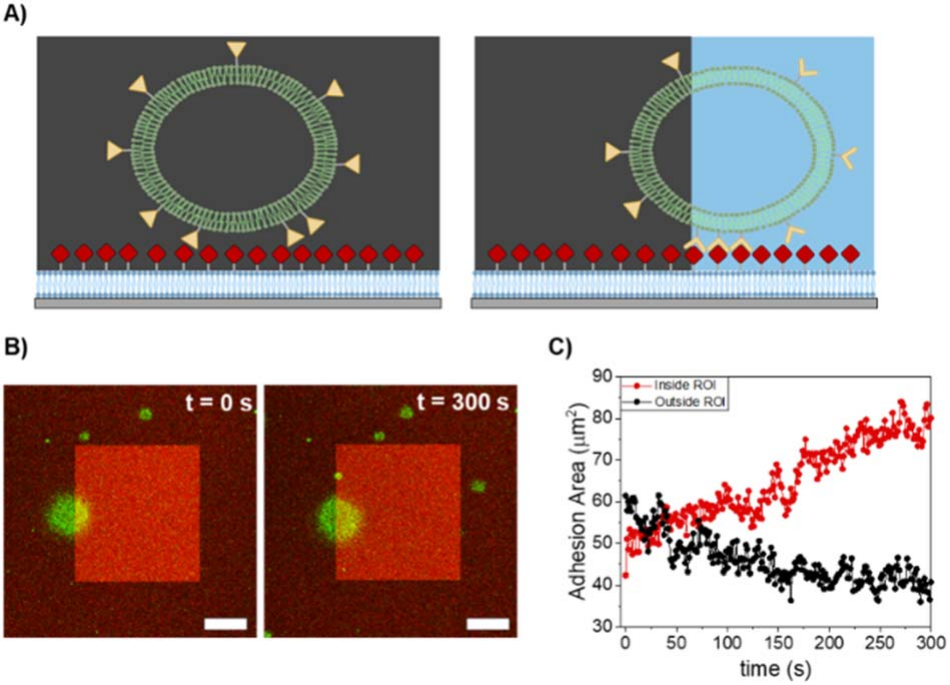
Light-induced motility of GUVs on SLBs. (A) Schematic representation of the light-dependent motility of GUVs on SLBs with high mOrange-nano density. (B) Confocal microscopy scans of the adhesion areas of iLID-functionalized GUV (0.25 mol% DGS-NTA) on nano-functionalized SLBs (10 mol% DGS-NTA) during illumination cycles with blue light. Adhesion are generated in the illuminated area (ROI, rapresented with a bright red square) that enables the movement of the GUVs towards the ROI. Scale bar represents 10 μm. (C) Plot of the adhesion area of the GUV inside (red) and outside (black) the illuminated area *versus* time.

In contrast, when the same GUVs were placed on SLBs with 10% DGS-NTA a clear, light-guided movement towards the illuminated region was observed (Fig. 5A, S10 and Supplementary Video). Within approximately 5 minutes, the adhesion area in the illuminated ROI progressively expanded, while the GUV area outside the illuminated area exhibited a corresponding decrease, suggesting an extension-retraction type of GUV motion on the surface (Fig. 5B). Across all replicates, the average migration speed of the GUVs was 0.4 μm min^−1^. However, attempts to further guide the GUV by shifting the illuminated region did not result in continued movement. This lack of responsiveness to subsequent ROI is likely due to the irreversible nature of the adhesions formed at higher ligand densities, which prevents detachment and re-initiation of new adhesions. Importantly, motility was observed exclusively in deflated GUVs, underscoring the essential roles of membrane fluctuations and the formation of local membrane protrusions in establishing new adhesions within the illuminated area, while the GUV rear can contract.

These experiments demonstrate that both ligand density and surface mobility are critical factors for inducing GUV motility on SLBs. On surfaces with low density and high mobility, light-dependent adhesion could be triggered, but GUV movement could not be achieved. We attribute this lack of motility not only to the limited number of ligand-receptor interactions formed in the illuminated area, but also to the dynamic recruitment of ligands beneath the GUV adhesion site. Due to the high mobility of ligands within the SLBs, local enrichment occurs at the adhesion zone, which compromises the desired asymmetry in adhesions necessary for directional movement. To overcome this, a reduction in ligand mobility is required. In our setup, this was possible by increasing the ligand density on the SLB. Under these conditions, ligand mobility was sufficiently restricted to establish asymmetry in the adhesions, permitting GUV movement in the illuminated area, in agreement with our previous study using immobile PEG-modified substrates. However, on these SLBs with high ligand density, further movements after the first step were not possible, as the formed adhesions were irreversible, which ultimately halts GUV progression. Overall, these findings suggest that sustained GUV motility requires a delicate balance: ligand mobility must be low to prevent clustering-induced irreversibility, but ligand density must also remain below a critical threshold to avoid irreversible adhesion. Only under such conditions can the adhesion asymmetry necessary for repeated and directional movement of synthetic cells be maintained. In other words, these findings suggest that a more efficient synthetic cells motility can be generally achieved on immobile surfaces such as PEG-modified substrates or on gel-state SLBs, on which the ligand density can be precisely tuned. Although the speed of the GUVs depends on several conditions, such surfaces showed that GUVs could reach 4.9 μm min^−1^ speed.^21^ Alternatively, improved motility can be also achieved by reducing the ligand-receptor interaction strength. However, light-dependent binding pairs should be accurately selected based both on interaction affinity and kinetics, as rapid deactivation is fundamental to generate faster motion.

## Conclusions

In this study, we systematically investigated how the surface density and lateral mobility of ligands on SLBs influence light-controlled adhesion and motility of synthetic cells. Using the photoswitchable interactions of iLID and nano, we demonstrated that both ligand density and receptor density critically determine the strength, reversibility, and dynamics of adhesions formed at the GUV-SLB interface. At low ligand densities with high mobility, adhesion remained light-dependent and reversible. However, local recruitment of mobile ligands led to ligand-receptor clustering at the adhesion sites, resulting in irreversible binding under certain conditions. In contrast, high ligand densities restricted ligand mobility and suppressed clustering, allowing directional GUV motility upon localized illumination, though subsequent motion was limited due to irreversible adhesion build up.

Our findings reveal a key design principle for synthetic cell motility; effective movement on lipid surfaces with mobile ligands requires a careful balance between ligand density, mobility, and adhesion strength. In particular, adhesion-based motility can fail on such dynamic surfaces due to ligand-receptor clustering and may only succeed when ligand mobility is sufficiently restricted. When a living cell crawls over other cells, the adhesion ligands on the underlying cells may be immobilized through cytoskeletal coupling. Alternatively, migrating cells can switch to adhesion-independent, amoeboid-like movement. These insights provide a foundation for the future engineering of cell-like systems that navigate using adhesion-based migration strategies.

## Author contributions

D. D. I and S. V. W. conceived the project. D. D. I. performed the experiments and analysed the data, and A. H. assisted in the optimization of GUV preparation and functionalization. S. V. W. supervised the project. D. D. I. and S. V. W. wrote the paper.

## Conflicts of interest

There are no conflicts to declare.

## Supplementary Material

SC-016-D5SC05324B-s001

SC-016-D5SC05324B-s002

## Data Availability

All other data will be made available upon reasonable request. The datasets supporting this article have been uploaded as part of the SI. See DOI: https://doi.org/10.1039/d5sc05324b.
