# Supplementary material for: Design rules for adhesion-driven synthetic cell motility on dynamic membranes
Source: Chem Sci. 2025 Aug 19;16(36):16638–44. doi: 10.1039/d5sc05324b (PMC12378535; doi:10.1039/d5sc05324b)
Supplement: SC-016-D5SC05324B-s002 [file SC-016-D5SC05324B-s002.pdf]

Supplementary Information (SI) for Chemical Science.
This journal is © The Royal Society of Chemistry 2025

Supporting Information

Design Rules for Adhesion-Driven Synthetic Cell Motility on Dynamic Membranes

Daniele Di Iorio, Ali Heidari and Seraphine V. Wegner*

Materials and Methods

Materials

Lipids 1-palmitoyl-2-oleoylphosphatidylcholine (16:0−18:1 PC) (catalog number: 850457), 1-palmitoyl-2-oleoyl-sn-glycero-3-phos-pho-(1′-rac-glycerol) (16:0−18:1 PG) (catalog number: 840457), and 1,2-dioleoyl-sn-glycero-3-(N-(5-amino-1-carboxypentyl)-iminodiacetic acid) succinyl) Ni^{2+}-salt (18:1 DGS-NTA(Ni)) (catalog number: 790404) were purchased from Avanti Polar Lipids in chloroform. The membrane dyes 1,1′-dioctadecyl-3,3,3′,3′-tetramethylindocarbocyanine perchlorate (DiL) (catalog numberD282-100 mg) and 1,1′-dioctadecyl-3,3,3′,3′-Tetramethylindodicar-bocyanine (DiD) (catalog number D7757-10 mg) were purchased from Thermo-Fisher Scientific. All microscopy experiments for GUV were performed in μ-slide 18-well glass bottom chambers from ibidi (catalogue number 81817). pQE-80L iLID (C530M) and pQE-80L MBP-SspB Nano were gifts from Brian Kuhlman (Addgene plasmid 60408 and 60409, respectively).[1]

iLID, Nano and mOrange-Nano purification

All proteins were expressed and purified as previously described.[2] The plasmids were transformed into *E. coli* BL21 (DE3) cells and a 10 mL overnight culture was grown in Luria Bertani (LB) medium supplemented with 50 μg/mL ampicillin at 37°C and 200 rpm. The culture was then inoculated into a fresh 1 L LB medium containing 50 μg/mL ampicillin and grown at 37 °C and 200 rpm until the OD600 reached 0.6-0.8. Protein expression was induced with 500 μM IPTG and culture was incubated overnight at 16 °C and 200 rpm. The bacteria were harvested by centrifugation (6000 rpm, 4 °C, 8 min), resuspended in 20 mL Buffer A (300 mM NaCl, 50 mM Tris, pH=7.4) supplemented with 100 mM PMSF, and were lysed by sonication. The lysate was cleared by centrifugation (12000 rpm, 4 °C, 30 min) and the supernatant was loaded onto a Ni^{2+}-NTA affinity column (HiLoad 16/600 Superdex 200 pg, GE Healthcare), which was preequilibrated with Buffer A. The column was washed with 30 mL Buffer A with 12 mM imidazole and then eluted with 10mL Buffer A with 2250 mM imidazole. The purity of proteins was analyzed by SDS-PAGE and protein concentrations were determined by UV-Vis spectroscopy.

Small unilamellar vesicles (SUVs) formation

SUVs were prepared by following previously reported methods.[3, 4] Lipid solutions were formed by mixing of 1 mg/mL 1,2-Dioleoyl-sn-glycero-3-phosphocholine (DOPC) with the desired mol% of 1,2-dioleoyl-sn-glycero-3-(N-(5-amino-1-carboxypentyl)-iminodiacetic acid) succinyl) Ni^{2+}-salt (DGS-NTA(Ni)) in glass vials. Where needed 1% DiD was added for visualization using confocal fluorescence microscopy. Afterwards, chloroform was slowly evaporated with a nitrogen stream while rapidly rotating the vial in order to form a thin lipid film. The vial was subsequently placed under vacuum in order to remove the residual chloroform for at least 1 h under vacuum. The dried film was rehydrated in MilliQ water with a final concentration of 1 mg/mL lipids. The obtained opaque solution was sonicated for 10 min until the solution turned clear. The obtained SUV solutions were transferred to an Eppendorf tube and stored in the fridge and used within 1 week.

QCM-D measurements

All QCM-D measurements were performed using a Q-Sense E4 system (Q-Sense) on SiO_2 crystals (Q-sense). Measurements were performed at 23 ºC and operated with four parallel flow chambers. An Ismatec peristaltic pump was used with a flow rate of 75 µl/min. For every measurement, the 5th overtone was reported for the normalized frequency (Δf_5) and dissipation (ΔD_5). Before the measurements QCM-D crystals were immersed in a 2 wt% sodium dodecyl sulfate (SDS) solution for 30 min, thoroughly rinsed with Milli-Q water and dried under a nitrogen stream. Subsequently, the sensors were activated by placing them in an UV/ozone cleaner (Ossila) for 10 min. The activated sensors were placed in the QCM-D chambers and flushed with buffer (10 mM Tris pH 7.4, 100 mM NaCl) until a stable baseline was reached. For the formation of SLBs in QCM-D, freshly prepared SUVs were diluted to a concentration of 0.1 mg/mL in buffer supplemented with 10 mM $CaCl_2$. The quality of the formed SLBs was monitored for each experiment, where high quality SLBs are defined by $\Delta f = 24 \pm 1$ Hz and $\Delta D < 0.5\times10^{-6}$. The SLBs were functionalized consecutively with 1 µM His-tag Nano until a plateau value was reached. Between each step, buffer was flushed over the sensors to ensure the wash off the unbound lipids or proteins.

Supported lipid bilayers (SLBs) formation in well-plates

SLBs were formed in 18-well microslides (Ibidi) with a glass bottom. For the formation of the SLBs, 150 µL of aqueous 2 M sodium hydroxide solution was added to the glass substrate for 1 h to activate the surface. Afterward, the wells were rinsed three times with ultrapure water and three times with buffer (10 mM Tris, 100 mM NaCl, pH 7.4) containing 10 mM $CaCl_2$, leaving 150 µL of buffer in the wells. Subsequently, a 15 µL solution of 1 mg/mL SUVs in water was added to the well (final SUV concentration 0.1 mg/mL) for 30 min at room temperature in order to generate a Ca^{2+}-induced rupture of the SUVs.[5] The SLBs were formed by the rupture of SUVs onto the glass substrate. The excess lipids were removed from the well by rinsing with 80 µL buffer without $CaCl_2$ at least five times. After the SLB formation, 100 µL buffer was left in the well plate in order

to preserve the SLBs. Afterwards, SLBs were functionalized with mOrange-Nano by adding protein solutions directly in the wells, obtaining 1 µM of final protein concentration.

FRAP measurements

All FRAP measurements were performed with a Leica SP8 confocal laser scanning microscope through a 63× water objective. For the FRAP experiments, SLBs containing varying mol% of DGS-NTA lipids were formed as described above. With a confocal microscope, a circular spot of ~14 µm in diameter was bleached by using 552 and 638 nm laser at 100% intensity, and the fluorescence intensity in the bleached regions was monitored over time. The FRAP protocol consisted of 5 pre-bleaching scans (0.369 s intervals), 5 bleaching scans (0.369 s intervals), and 40 post-bleaching scans (10 scans at 0.369 s intervals, followed by 30 scans at 1 s intervals, and 10 additional scans at 10 s intervals). The mOrange protein was excited with a 552 nm laser, and the emission was detected at 580-625 nm; DiD dye was excited with a 638 nm laser, and the emission was detected at 650–700 nm. All images were analyzed by using Leica Application Suite X (LAS X) software version 3.7.1.21655. The fluorescence recovery profile was fitted with Origin, using an exponential decay function (ExpDec1). From the fitting we derived the half-lives ($t_{1/2}$) of fluorescence recovery, and calculated the apparent diffusion coefficient (D) by the formula $D = (0.88\omega^2)/4 \cdot t_{1/2}$, where ω is the radius of the bleached ROI.

Giant unilamellar vesicles preparation and functionalization

GUVs were prepared using the assisted gel GUV formation method, as previously described.[2] A 5% (w/v) polyvinyl alcohol (PVA) (MW: 145 000 g/ mol) solution with 100 mM sucrose was prepared in Milli-Q water overnight at 80 °C at 400 rpm. Then, 40 µL of PVA solution was spread as a thin layer on top of a 60 x 24 mm glass slide and dried at 50 ºC for 30 min. Afterwards, 5 µL of a lipid solution (10 mg/mL POPC, 10 mol% 1-palmitoyl-2-oleoyl-sn-glycero-3-phospho-(1'-rac-glycerol) (POPG), and 0.1mol%, 0.25mol% or 0.5mol% DGS-NTA and 1 mol% DiD) in chloroform were spread and dried on the PVA layer at 30 ºC. Subsequently, a chamber was assembled on the functionalized slide using a Teflon spacer (ca. 40 mm × 24 mm × 2 mm) and a second glass slide. The GUVs were formed by adding 1 mL of rehydration buffer (10 mM Tris pH 7.4, 100 mM NaCl) into the chamber for 1 hour at room temperature. The chamber was inverted, gently taped twice using a pipet tip and the GUVs were harvested into a 1.5 mL LoBind Eppendorf tube. To functionalize the outer membrane of the GUVs with His-tagged iLID or mOrange-Nano protein, 100 µL of GUV solution was taken from the bottom of the plastic tube and collected in a new plastic tube, where protein was added in solution with a final concentration of 100 nM. The solution containing protein decorated GUVs was kept for 30 min in the dark with lid open to enable deflation of GUVs. Subsequently, 5 µL of deflated GUVs solution were added to the SLB-functionalized ibidi wells filled with 150 µL of buffer for further analysis, allowing for a 30-fold dilution of the GUV solution and thus minimalizing the effect of unbound iLID. The sample was placed in the confocal microscope for approximately 30 min in the dark to allow GUVs to settle at the bottom of the wells.

Imaging of light-dependent adhesion of GUVs

For the GUVs imagining, a 63x water objective was used, and the 633 nm laser was used to image both the lipid dye DiD and the membrane. Deflated GUVs were selected and a ROI was delineated to illuminate the designated GUVs with blue light (488 nm, 1% intensity). GUVs were imaged at the (x,z) cross-section and and illuminated continuously with blue light for ca 15 min. The GUV were always imaged at the position of maximum diameter. Subsequently, the sample was imaged without blue light illumination for ca 15-20 more minutes to investigate adhesion reversibility. The measurements were stopped when no adhesion or reversion was observed. Imaged were analyzed using imageJ, and the adhesion of the GUV at the SLB interfaces (x,z cross-section) was quantified for each time point. Adhesion area was then calculated assuming a spherical area of a GUV contact area on the SLB.

Light-induced motility of GUVs

To observe and quantify the GUVs motility, the GUVs were observed and imaged in x,y plane in correspondence to the SLB. A region of interest (ROI) was chosen such that approximately half of the GUV adhesion area was included in the illuminated area, and the second half was not illuminated. The ROI was then illuminated continuously with blue light (488 nm Laser) at 3% laser intensity and the GUV's movement was monitored only at the adhesion area by acquiring 1 scan every 1.3 s using both the 552 and the 633 nm laser light excitation to measure simultaneously the SLBs (DiL) and the GUVs (DiD), respectively. The measurements were conducted until the GUV stopped moving into the illuminated area. To analyse the movement of GUV, images were first binarized with ImageJ, setting the same threshold values for all time point images. Subsequently, the GUV center of mass was determined for the initial and final time point using the analyse particle tool in ImageJ. From the obtained coordinates, the GUV displacement was calculated and divided by the corresponding time intervals.

References

1. Guntas, G.; Hallett, R. A.; Zimmerman, S. P.; Williams, T.; Yumerefendi, H.; Bear, J. E.; Kuhlman, B., Engineering an improved light-induced dimer (iLID) for controlling the localization and activity of signaling proteins. *Proceedings of the National Academy of Sciences* **2015,** *112* (1), 112-117.
2. Bartelt, S. M.; Chervyachkova, E.; Steinkühler, J.; Ricken, J.; Wieneke, R.; Tampé, R.; Dimova, R.; Wegner, S. V., Dynamic blue light-switchable protein patterns on giant unilamellar vesicles. *Chemical Communications* **2018,** *54* (8), 948-951.
3. Di Iorio, D.; Bergmann, J.; Higashi, S. L.; Hoffmann, A.; Wegner, S. V., A disordered tether to iLID improves photoswitchable protein patterning on model membranes. *Chemical Communications* **2023,** *59* (29), 4380-4383.
4. Di Iorio, D.; Wegner, S. V., Dynamic Light-Induced Protein Patterns at Model Membranes. *JoVE* **2024,** (204), e66531.
5. Richter, R. P.; Bérat, R.; Brisson, A. R., Formation of Solid-Supported Lipid Bilayers: An Integrated View. *Langmuir* **2006,** *22* (8), 3497-3505.

Supporting Figures

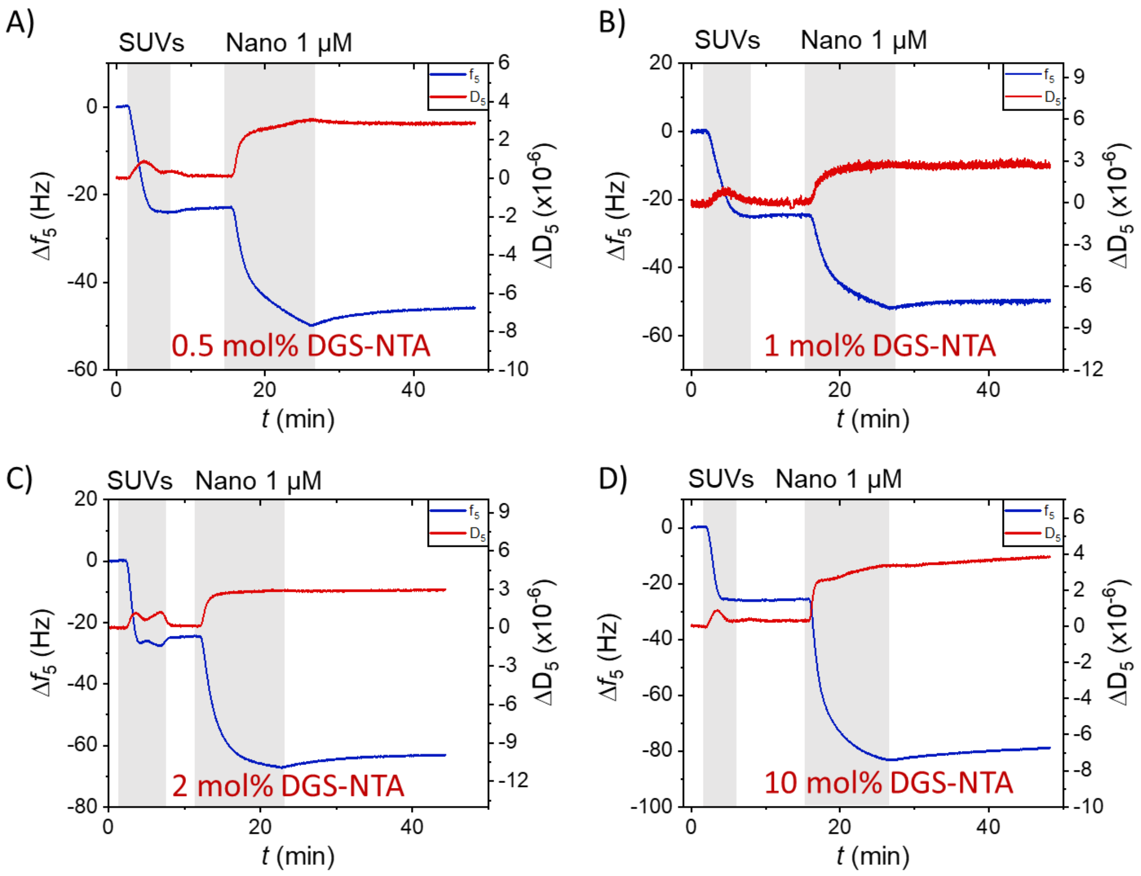

Figure S1: QCM-D measurements showing stepwise the formation of an SLB using DOPC-based SUVs doped with 0.5 mol% (A), 1 mol% (B), 2 mol% (C) and 10 mol% (D) DGS-NTA(Ni^{2+}) lipids, and the subsequent binding of His-tagged Nano. Gray shadings indicate the addition of indicated components, while white areas indicate washing steps with buffer.

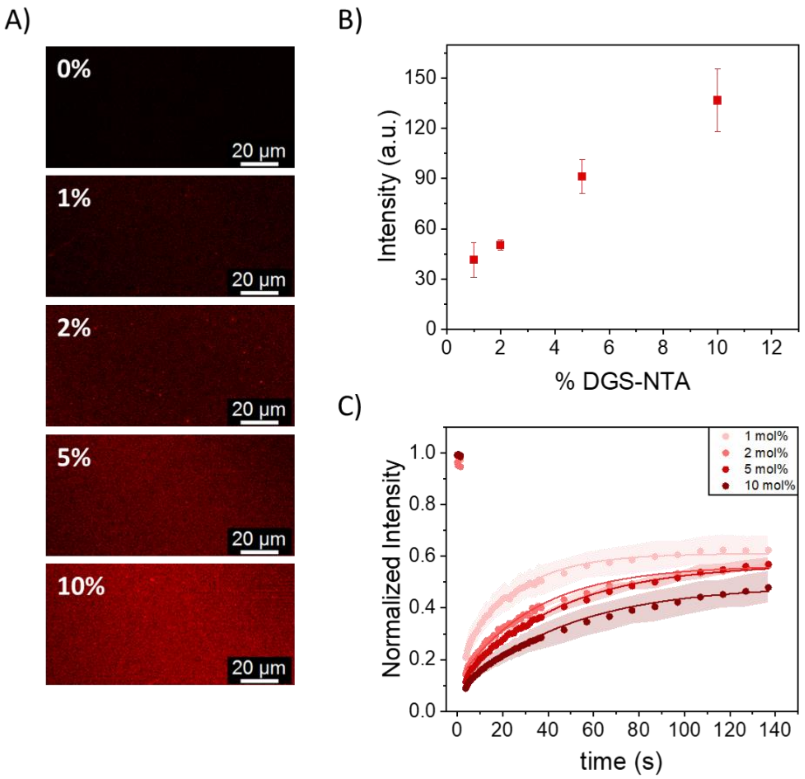

D)

DGS-NTA	Diffusion constant ($\mu m^2/s$)
1 %	0.68
2 %	0.47
5 %	0.37
10 %	0.34

Figure S2: (A) Example of fluorescence microscopy images of SLBs containing increasing % DGS-NTA lipids and functionalized with 1 µM mOrange-Nano. (B) Quantification of fluorescence intensity of mOrange-Nano on SLBs containing varying % of DGS-NTA lipid obtained from the fluorescence images.

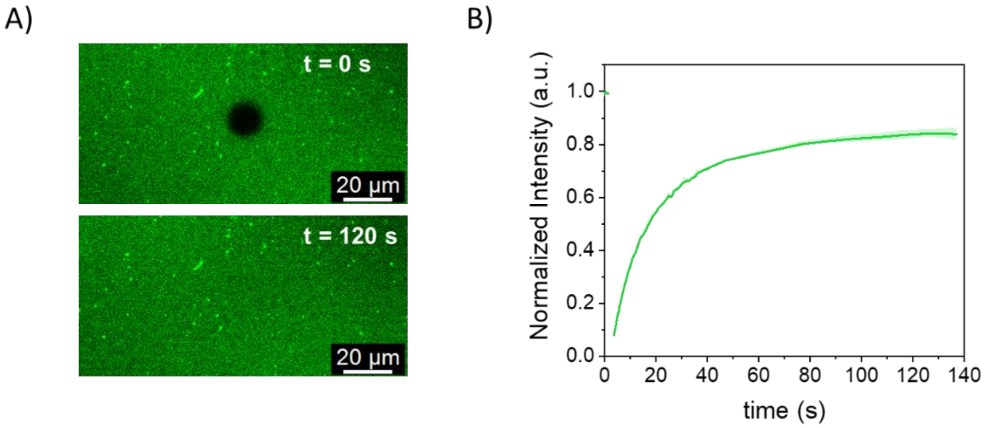

Figure S3: FRAP measurement on SLBs containing DiD fluorescent dye. (A) Fluorescence images of SLBs containing 0.1% DiD and 1% DGS-NTA immediately after photobleaching (t = 0 s) and after recovery (t = 120 s). (B) Recovery of DiD fluorescence intensity in time after photobleaching.

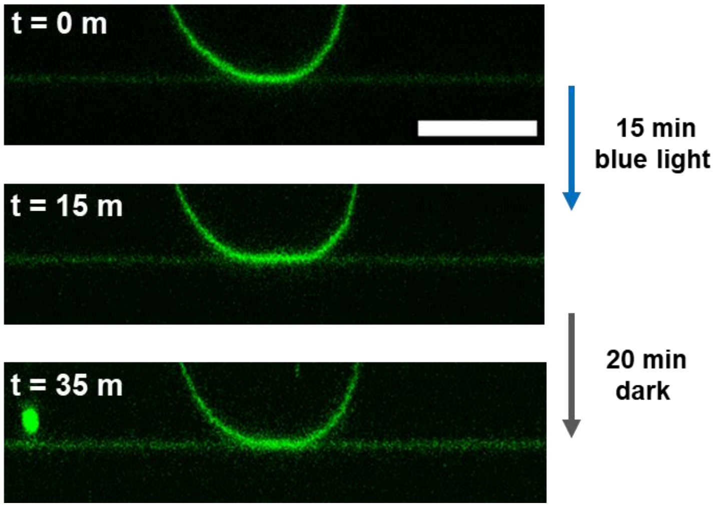

Figure S4: Fluorescence microscopy images showing the contact area of a GUV containing 0.25% DGS-NTA and functionalized with iLID adhering on SLBs doped with 1 mol% DGS-NTA and functionalized with Nano (zoom-in of Fig. 3A).

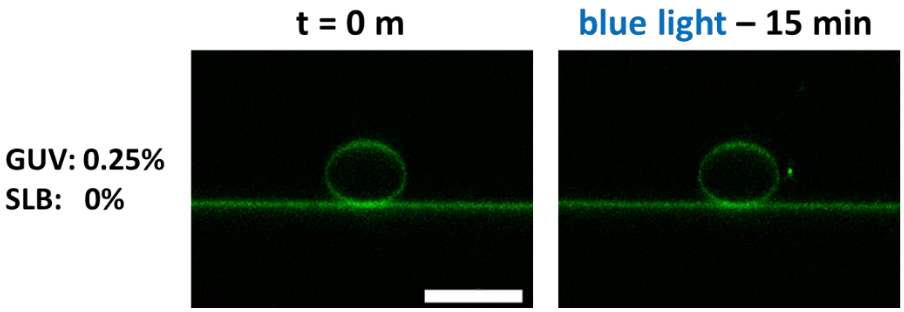

Figure S5: Fluorescence microscopy images iLID-GUV with 0.25% DGS-NTA not adhering to unfunctionalized SLB. Scale bar = 20 µm.

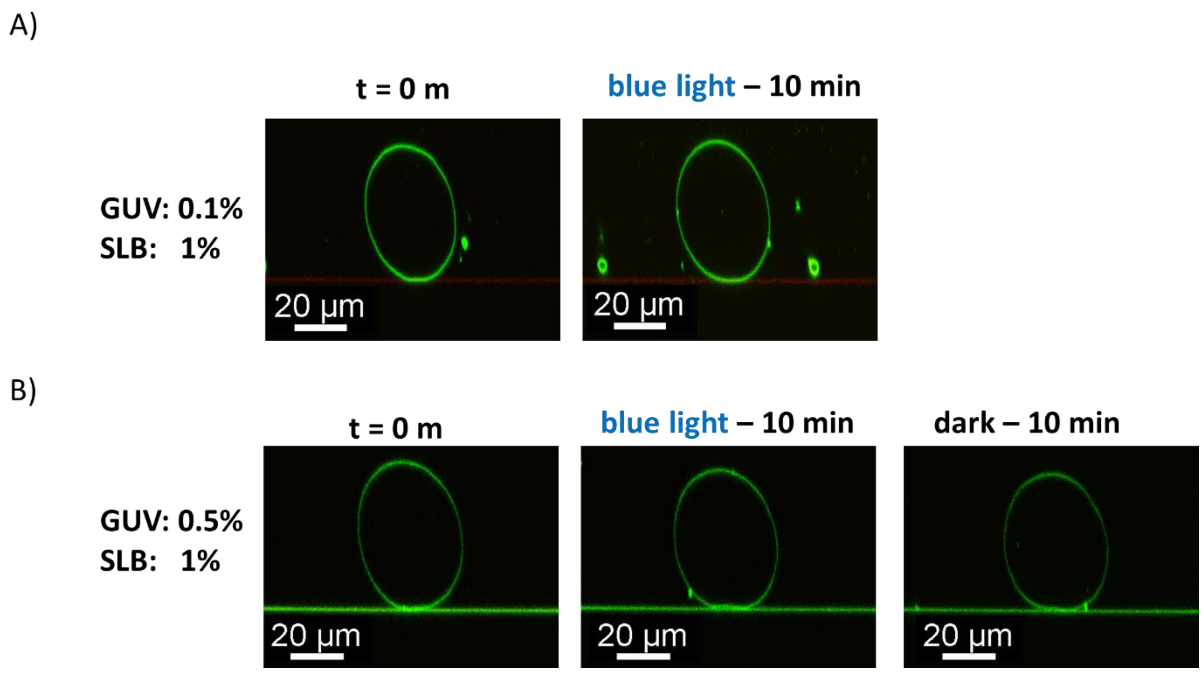

Figure S6: Fluorescence microscopy images of iLID-GUV with 0.1% (A) and 0.5% (B) DGS-NTA on Nano-functionalized SLBs doped with 1% DGS-NTA.

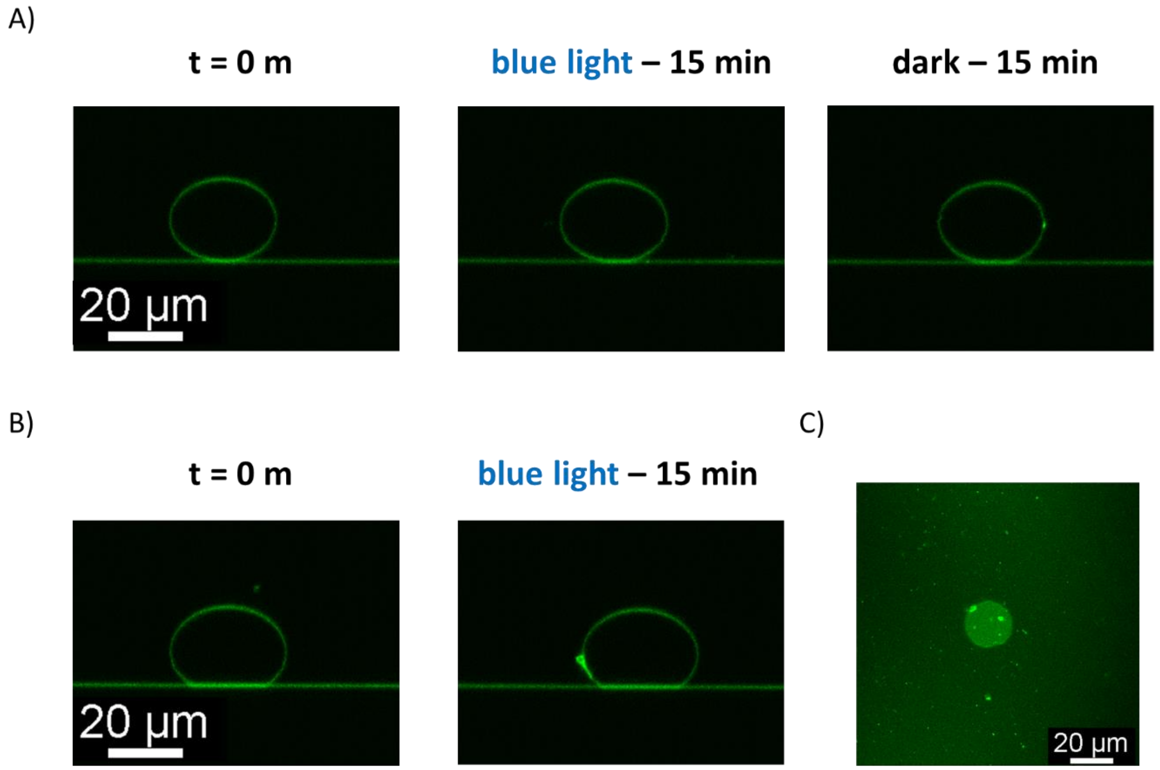

Figure S7: Fluorescence microscopy images iLID-GUV with 0.25% (A) and 0.5% (B) DGS-NTA on Nano-functionalized SLBs with 10% DGS-NTA. C) Adhesion area of GUV in (B) after 15 min of blue light illumination.

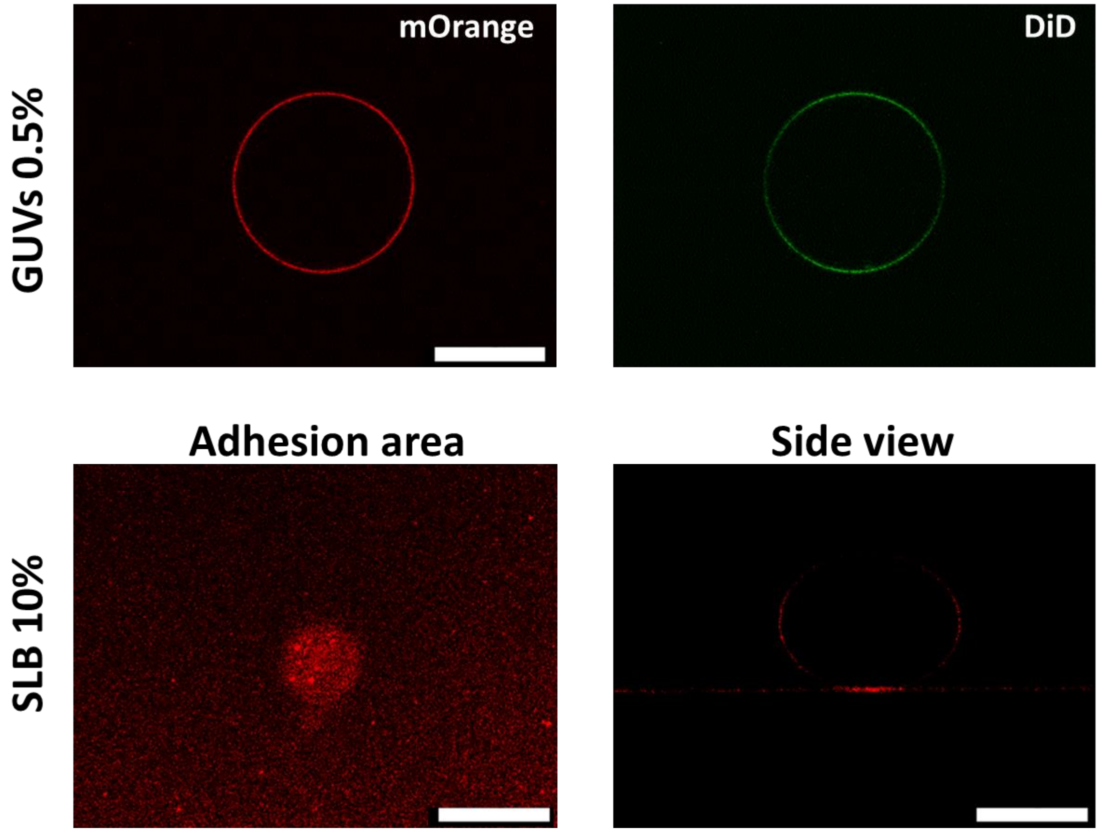

Figure S8: Fluorescence microscopy images GUV containing 0.5% DGS-NTA and functionalized with mOrange-Nano adhering on iLID-functionalized SLBs with 10% DGS-NTA. The higher mOrange intensity observed in the adhesion area on the SLBs indicates recruitment of mOrange-Nano upon the light-induced adhesion of the GUV.

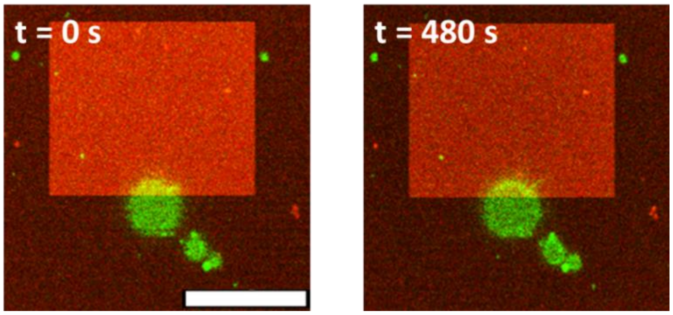

Figure S9: A) Confocal microscopy scans of the adhesion areas of iLID-functionalized GUV (0.25 mol% DGS-NTA) on Nano-functionalized SLBs (1% DGS-NTA) during illumination with blue light, generating no GUV motility. Scale bar represents 20 µm.

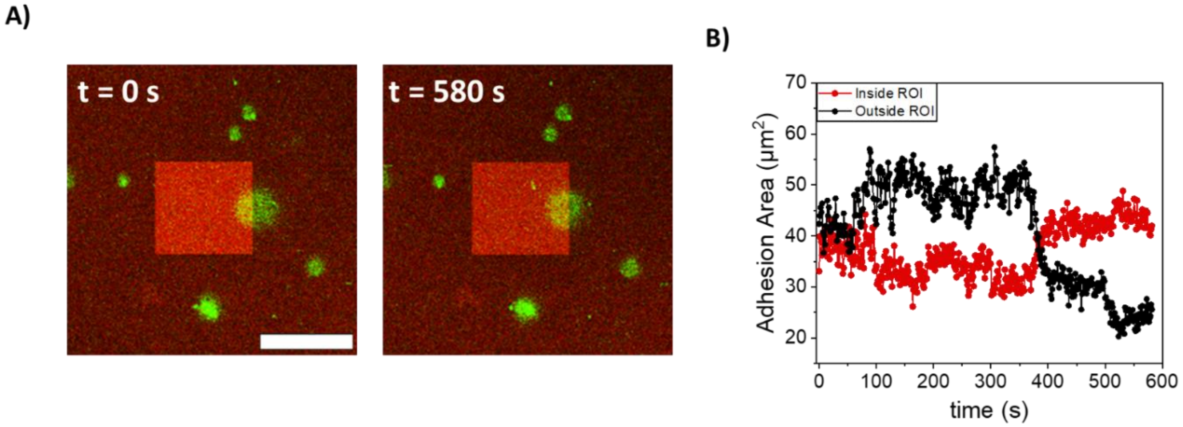

Figure S10: A) Confocal microscopy scans of the adhesion areas of iLID-functionalized GUV (0.25% DGS-NTA) on Nano-functionalized SLBs (10% DGS-NTA) during illumination with blue light. Adhesion area generated in the illuminated area (ROI, represented with a bright red square) that enables the movement of the GUVs towards the ROI. Scale bar represents 20 µm. B) Plot of the adhesion area of the GUV inside (red) and outside (black) the illuminated area versus time.
